# Differential Angiogenic Potential of 3-Dimension Spheroid of HNSCC Cells in Mouse Xenograft

**DOI:** 10.3390/ijms22158245

**Published:** 2021-07-31

**Authors:** So-Young Choi, Soo Hyun Kang, Su Young Oh, Kah Young Lee, Heon-Jin Lee, Sangil Gum, Tae-Geon Kwon, Jin-Wook Kim, Sung-Tak Lee, Yoo Jin Hong, Dae-Geon Kim, Su-Hyung Hong

**Affiliations:** 1 Department of Oral and Maxillofacial Surgery, School of Dentistry, Kyungpook National University, Daegu 41940, Korea; dentalchoi@knu.ac.kr (S.-Y.C.); kwondk@knu.ac.kr (T.-G.K.); vocaleo@knu.ac.kr (J.-W.K.); st0907@knu.ac.kr (S.-T.L.); yoojin.hong34@gmail.com (Y.J.H.); ktg4280@naver.com (D.-G.K.); 2 Department of Microbiology and Immunology, School of Dentistry, Kyungpook National University, Daegu 41940, Korea; black_bean@knu.ac.kr (S.H.K.); oohsuy@knu.ac.kr (S.Y.O.); christin_a@naver.com (K.Y.L.); heonlee@knu.ac.kr (H.-J.L.); 3 Binaree Co., Ltd., Daegu 41940, Korea; gold780807@hanmail.net

**Keywords:** oral squamous cell carcinoma, head and neck squamous cell carcinoma, spheroid, cancer-associated fibroblast, tissue clearing, angiogenesis, exosome

## Abstract

The experimental animal model is still essential in the development of new anticancer drugs. We characterized mouse tumors derived from two-dimensional (2D) monolayer cells or three-dimensional (3D) spheroids to establish an in vivo model with highly standardized conditions. Primary cancer-associated fibroblasts (CAFs) were cultured from head and neck squamous cell carcinoma (HNSCC) tumor tissues and co-injected with monolayer cancer cells or spheroids into the oral mucosa of mice. Mice tumor blood vessels were stained, followed by tissue clearing and 3D Lightsheet fluorescent imaging. We compared the effect of exosomes secreted from 2D or 3D culture conditions on the angiogenesis-related genes in HNSCC cells. Our results showed that both the cells and spheroids co-injected with primary CAFs formed tumors. Interestingly, vasculature was abundantly distributed inside the spheroid-derived but not the monolayer-derived mice tumors. In addition, cisplatin injection more significantly decreased spheroid-derived but not monolayer-derived tumor size in mice. Additionally, exosomes isolated from co-culture media of FaDu spheroid and CAF upregulated angiogenesis-related genes in HNSCC cells as compared to exosomes from FaDu cell and CAF co-culture media under in vitro conditions. The mouse tumor xenograft model derived from 3D spheroids of HNSCC cells with primary CAFs is expected to produce reliable chemotherapy drug screening results given the robust angiogenesis and lack of necrosis inside tumor tissues.

## 1. Introduction

Despite progress in the treatment of HNSCC including oral squamous cell carcinoma (OSCC), the 5-year survival rate has not substantially improved, remaining at 50–60% [1,2]. For early-onset HNSCC, surgical treatment is likely to yield an effective outcome. In recent years, HNSCC incidence in the elderly has increased due to population aging. In such cases, radiation therapy or chemotherapy may be preferred over surgical treatment depending on the patient’s overall health condition and quality of life considerations after surgery. In patients aged over 65 years, the use of chemoradiotherapy increased from 49% in 2000 to 82% in 2009 despite a lack of conclusive evidence regarding the effectiveness of this therapeutic intensification among elderly patients [3]. With anticancer drug use, toxicity and drug tolerance may differ depending on an individual’s systemic condition, and it is, therefore, difficult to select the appropriate anticancer drugs for each patient.

A three-dimensional tumor structure generates various physical and chemical gradients, which contribute to phenotypic heterogeneity within the tumor [4,5]. In vivo tumor growth occasionally contains a necrotic region that forms due to oxygen gradients and differential access to nutrients. Previous data showed that the development of a necrotic core in cancer patients is correlated with increased tumor size and poor prognosis, such as the emergence of chemoresistance [6]. Additionally, cell death via necrosis is not preferred as an anticancer strategy due to the consequent inflammatory response [7]. OSCC has different clinical characteristics from other solid tumors, one of which is that necrosis is observed in only about 10% of cases with a high grade of malignancy [8]. Also, when the clinicopathological features of 346 cases of OSCC were investigated, 76% were smaller than 4 cm, and only 6% were bigger than 6 cm [9]. As such, it is not common for OSCC tumors to grow large enough for central necrosis observation. Therefore, it is estimated that the effect of anticancer drugs on OSCC can be more accurately evaluated when the blood vessels are well-distributed around and inside the tumor, resulting in less necrosis.

Recently, ex vivo 3D culture has been widely applied to physiologically mimic the cancer microenvironment. Multicellular spheroids are one of the most commonly used models for 3D cell culture. In recent years, advances in 3D cell culture technology have allowed for uniform culture sizes with a high-throughput scale, which has proven useful for in vitro drug screening [10,11]. The use of 3D spheroids for constructing in vivo mouse tumor models is currently under evaluation. Massa et al. attempted to evaluate the available preclinical cancer models including 3D spheroids for personalized therapy and innovative therapeutic approaches [12]. Jung et al. co-cultured hepatocellular carcinoma cells with human umbilical vein endothelial cells to form spheroids, which induced angiogenesis and vessel maturation in vivo [13]. No studies to date have shown differences in tumor characteristics including angiogenesis between models constructed using 2D monolayer cells and 3D spheroids. Recent progress in cancer biology has substantially changed our understanding of the functional significance of cancer stroma. The tumor microenvironment plays a crucial role in neoplastic cell tumorigenesis, progression, and metastasis [14,15,16]. Cancer-associated fibroblasts (CAFs), one of the major cell components in the tumor microenvironment, reportedly participate in tumor progression. Additionally, CAFs promote in vivo cancer progression when co-injected with tumor cells or when recruited to the cancer formation site [17,18].

CAF is known to be essential in tissue homeostasis and is a key player in the process of tumorigenesis [3,19]. CAFs secrete growth factors and chemokines that alter the extracellular matrix (ECM) and oncogenic signals, thereby increasing the proliferation and invasion of cancer cells [1,12]. Moreover, CAFs promote cancer progression in vivo when co-injected with tumor cells or are recruited to the tumor site [13,14]. Interestingly, patient-derived CAFs could regulate cisplatin resistance in HNSCC cells via paracrine effects in in vitro conditions [19]. Furthermore, HNSCC cells interacting with CAF increase autophagy levels, contributing to cisplatin resistance [20]. Recent years have shown increased interest in personalized chemotherapy, and various attempts have been made to administer the most effective treatment to patients using animal cancer models injected or implanted with patient-derived tissues, but this strategy has been rare in OSCC, especially for CAFs.

Recently, extracellular vesicles known as exosomes have been recognized as crucial signaling mediators in regulating the tumor microenvironment. Exosomes are membrane-enclosed vesicles derived from the endosomal system during the formation of multivesicular bodies. Exosomes represent a novel mode of intercellular communication and contribute to a wide range of biological processes in health and disease such as cancer [21]. It is well known that exosomes comprise a variety of substances containing proteins, mRNAs, miRNAs, and lipids. These contents of exosomes regulate tumor growth, metastasis, and angiogenesis [22]. Ludwig et al. suggested that tumor-derived exosomes from HNSCC cell lines induce angiogenesis through reprogramming and modulation of endothelial cells [23]. Interestingly, Qin et al. showed that cisplatin treatment increases exosome secretion by CAFs and exosome-derived miRNA causes cisplatin resistance [24]. However, not much is known about the effect of tumor-derived exosomes on the HNSCC microenvironment. Here, we compared mouse tumors formed by HNSCC cells grown as 2D monolayers or 3D spheroids to develop an in vivo xenograft model. An attempt was also made to more accurately mimic the cancer microenvironment in xenograft formation by co-transplanting patient-derived CAFs. The effect of cisplatin was compared in nude mice given tumors derived from FaDu and primary HNSCC monolayer cells or spheroids with CAF. In addition, an attempt was made to confirm whether the tumor-derived exosomes are involved in angiogenesis in the cancer microenvironment.

## 2. Results

### 2.1. Characterization of Fibroblast Cells Cultured from HNSCC Tissues

**Figure 1:**
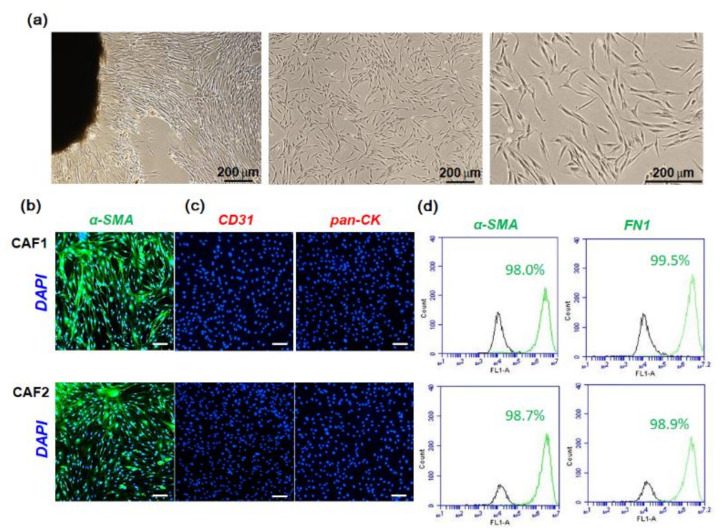
Immunocytochemical analysis of fibroblasts cultured from HNSCC patients. The stroma adjacent to the tumor was cut into the smallest possible pieces in sterile DMEM and seeded in 10-cm tissue culture dishes supplemented with 10% FBS and then cultured for 2–3 weeks. (**a**) The representative image was taken by phase-contrast microscope. (**b**,**c**) Cells were seeded in 6-well plates containing cover slides and immunostained with antibodies specific for fibroblasts (α-SMA), endothelial cells (CD-31), and epithelial cells (pan-cytokeratin). The white scale bars represent 100 μm. (**d**) α-SMA-positive or fibronectin (FN1)-positive fibroblasts were analyzed with a flow cytometer. The experiments were performed three times and representative images are shown.

After 2–3 weeks of incubation, fibroblasts attached to the culture plate began to grow out. All primary cultured fibroblasts had a homogeneous, spindle-shaped fibroblastic morphology. Representative microscopic images are shown in Figure 1a. α-SMA-expressing fibroblasts are considered the main constituents of stroma in various cancers [25]. Figure 1b shows the immunostained image of fibroblast cells with fluorescent α-SMA-antibody. To check for epithelial and endothelial cell contamination within the isolated CAF population, we examined the expression of pan-cytokeratin and CD31 (platelet endothelial cell adhesion molecule-1, PECAM-1). As shown in Figure 1c, the primary CAF cells were negative for both these markers. To further evaluate the CAF cells, α-SMA- or fibronectin (FN1)-stained cells were analyzed with a flow cytometer. More than 98% of the CAF cells showed positive expression of these two fibroblast markers (Figure 1d). The expression patterns of these markers persisted after several passages.

### 2.2. Mouse Tumorigenesis from FaDu Monolayer Cells and 3D Spheroids

**Figure 2:**
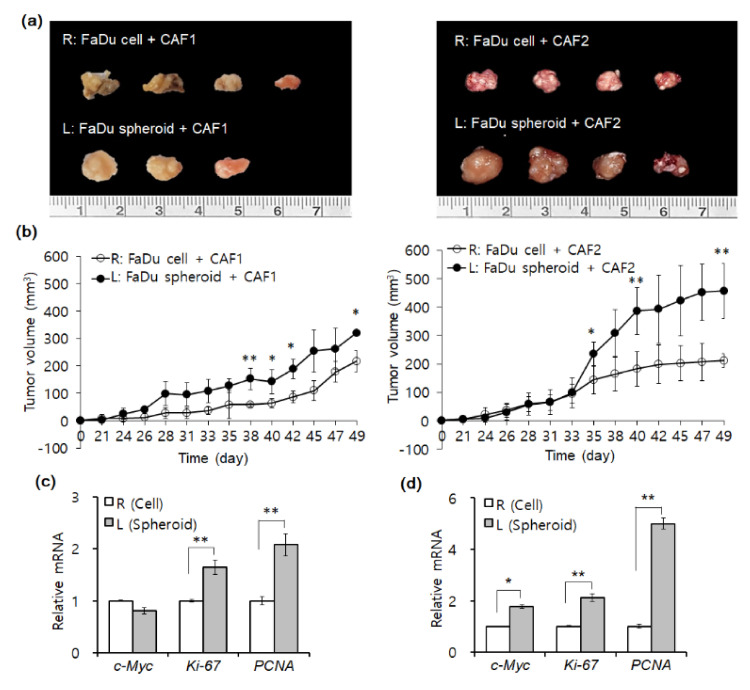
Mouse tumor xenograft formation from FaDu monolayer cells or 3D spheroids with patient-derived fibroblasts (CAF1 or CAF2). (**a**) FaDu spheroids (approximately 400 μm in diameter) were prepared in 96-well U-bottom ultra-low attachment plates. FaDu monolayer cells (5 × 105) and 50 FaDu spheroids (approximately 5 × 105 cells) were mixed with fibroblast cells (5 × 105), respectively, followed by injection into the oral mucosa of the right and left cheek of mice. This experiment was performed on five mice. (**b**) Twenty days after cell or spheroid injection with primary CAFs, average tumor volume was measured using a caliper. (**c**,**d**) mRNA expression (fold change) of some representative genes related to cell proliferation (c-Myc, Ki-76, PCNA) was analyzed in mice tumors by qPCR. Results represent the mean ± standard deviation. (* *p* < 0.05, ** *p* < 0.01).

We evaluated tumor formation upon injection of FaDu monolayer cells or 3D spheroids in the oral mucosa of the right or left cheek of mice, respectively. Two populations of primary CAFs (CAF1, CAF2) isolated from different HNSCC tumor tissues were co-injected with FaDu monolayer cells or spheroids into mice. The size of tumors formed during the same period by FaDu spheroids (Figure 2a,b, left cheek) showed a significant increase of size as compared to FaDu cells (Figure 2a,b, right cheek) in both CAFs. Only FaDu spheroid-derived tumors showed a remarkable difference in tumor size according to different CAF group cells (Figure 2b, left cheek). When we analyzed the mRNA expression of some cell proliferation-related genes (*c-Myc*, *Ki-76*, and *PCNA*) in mice tumors with CAF1 (Figure 2c) or CAF2 cells (Figure 2d), these genes showed significantly higher mRNA expression in tumors formed with FaDu spheroids.

**Figure 3:**
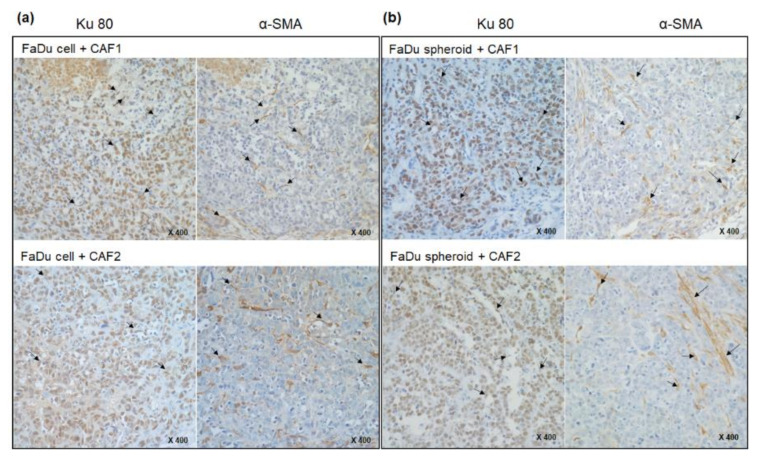
Immunohistochemical analysis of mice xenograft tumors tissues derived from FaDu cells (**a**) or spheroids (**b**) with primary CAFs. Tissues were immunostained with anti-Ku80 and α-SMA antibody. Ku80 biomarkers are broadly stained in tumor tissues, such as epithelial cells and fibroblasts (black arrows). Fibroblast-specific α-SMA staining was also detected inside the epithelial tumor cells (black arrows).

Immunohistochemical staining was performed to evaluate how human cells and CAFs were distributed in mice tumor tissues. Figure 3 shows that human species-specific Ku80 biomarkers are broadly stained in tumor tissues such as epithelial cells and fibroblasts, suggesting that FaDu and CAF cells injected into the mice cheeks were well involved in tumor xenograft formation. Fibroblast-specific α-SMA staining was also detected inside the epithelial tumor cells (Figure 3).

### 2.3. Angiogenesis of Mouse Xenograft from FaDu Monolayer Cells and 3D Spheroids

**Figure 4:**
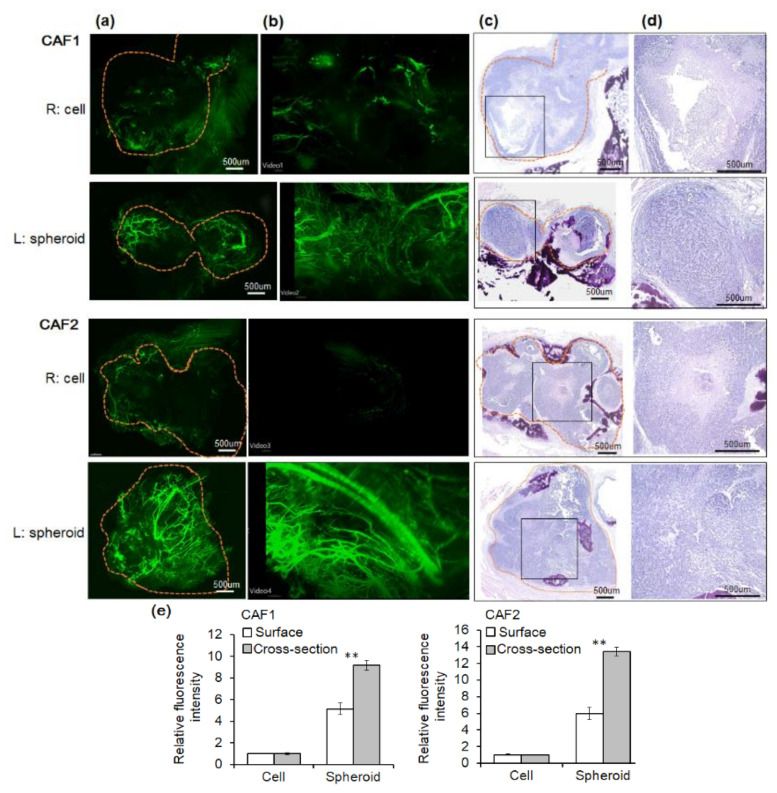
Comparison of blood vessel formation and H&E staining among tumor tissues. The tumor tissues were cut in half, after which vascular staining and H&E staining were performed. The images of vascular and H&E staining were compared in parallel. For vascular staining, mice were given an intracardiac injection of lycopersicon esculentum lectin and subsequently underwent complete perfusion. Tumor tissues were cleared and incubated in mounting and storage solution for 24 h. Blood vessel formation inside tumor tissues was evaluated using a Lightsheet Z.1 fluorescence microscope (×40). (**a**) Surface fluorescent images for blood vessels. The yellow dot lines represent the tumor margin in the mice tissues section. (**b**) The representative cross-section image of tumor tissue in (**a**). (**c**) H&E staining images of tumor tissues were compared to evaluate necrosis (×40). (**d**) A magnified image of the rectangle in (**c**) (×100). (**e**) Fluorescent vascular staining was quantified from tumor surfaces and the cross-sectioned images were extracted every 5 s from video of the cleared tissue (** *p* < 0.01).

Next, we compared blood vessel formation in tumor tissues formed in both the right and left cheeks. Some blood vessels were observed around the tumor arising from monolayer cells with CAF co-injection (Figure 4a,e), but there was little vascular distribution within the tumor (Figure 4b,e), (Supplementary Videos S1 and S3). In contrast, co-injection of spheroids with CAFs increased both the vascular distribution around and inside the tumor, as well as tumor cellularity (Figure 4a,b,e), (Supplementary Videos S2 and S4). Figure 4c,d are the H&E staining images of tumor tissues. As shown in Figure 4d, an enlarged rectangular image of 4c, cell-derived tumor tissues showed remarkably increased necrosis compared to spheroid-derived ones. We further analyzed the mRNA expression of some representative angiogenesis-related genes in FaDu cell or spheroid with CAF co-culture by in vitro Transwell system. Interestingly, CAF co-culture upregulated mRNA expression which promoting angiogenesis specifically in spheroid derived from FaDu or primary OSCC cells as compared to monolayer cells (Supplementary Figure S1). There was no remarkable difference in the mRNA level in FaDu monolayer cells according to CAF co-culture.

### 2.4. Effect of Cisplatin on Mice Tumor from FaDu and Primary HNSCC Cells

**Figure 5:**
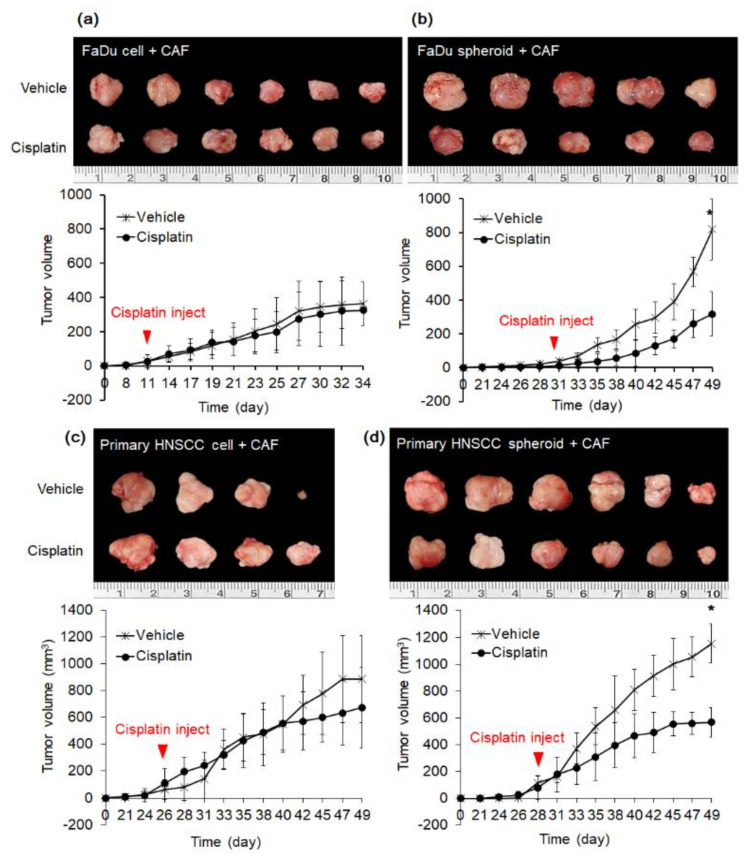
Effect of cisplatin on tumors in nude mice derived from FaDu and primary HNSCC cells. (**a**,**b**) FaDu monolayer cells (5 × 105) or 50 spheroids (approximately 5 × 105 cells) were co-injected with the same number of CAF cells into both cheeks in mice oral mucosa. Cisplatin was injected from the time the tumor was observed for 18–23 days. (**c**,**d**) Primary HNSCC monolayer cells (5 × 105) and 50 spheroids (approximately 5 × 105 cells) were co-injected with the same number of CAF cells into both cheeks in mice oral mucosa. After 25–27 days, tumor formation was observed and cisplatin was injected for 3 weeks. The dissociated cell group and the spheroid group consisted of eight mice each. After tumor formation, each group was divided into two groups including cisplatin injection (n = 4 with transplantation in both cheeks) and PBS vehicle injection control (*n* = 4 with transplantation in both cheeks). Cisplatin (2.5 mg/kg) or vehicle control was intraperitoneally injected 3 times a week and sacrificed on the 18–23th day after cisplatin administration. Results represent the mean ± standard deviation (* *p* < 0.05).

We further compared the effect of cisplatin in mice xenograft derived from spheroids or dissociated cells. We conducted this experiment on FaDu cells and primary HNSCC cells cultured from patients. As shown in Figure 5a, mice tumors derived from FaDu cell and CAF2 showed moderate proliferation when they were observed after 10 days of transplantation. On the contrary, when FaDu spheroid was transplanted with CAF, the tumor was detected after 30 days, but after that, tumor volume increased rapidly (Figure 5b). Mice tumors derived from primary HNSCC cells or spheroids with CAF2 were observed around the same time after 25–27 days of transplantation (Figure 5c,d). Mice were injected with cisplatin or vehicle control for 18–23 days according to the bodyweight or body condition. The tumor growth curves of each group demonstrate that tumors derived from spheroid significantly decreased in volume upon cisplatin treatment in both tumor models. In contrast, there was no significant effect of cisplatin treatment in tumors derived from monolayer cancer cells.

**Figure 6:**
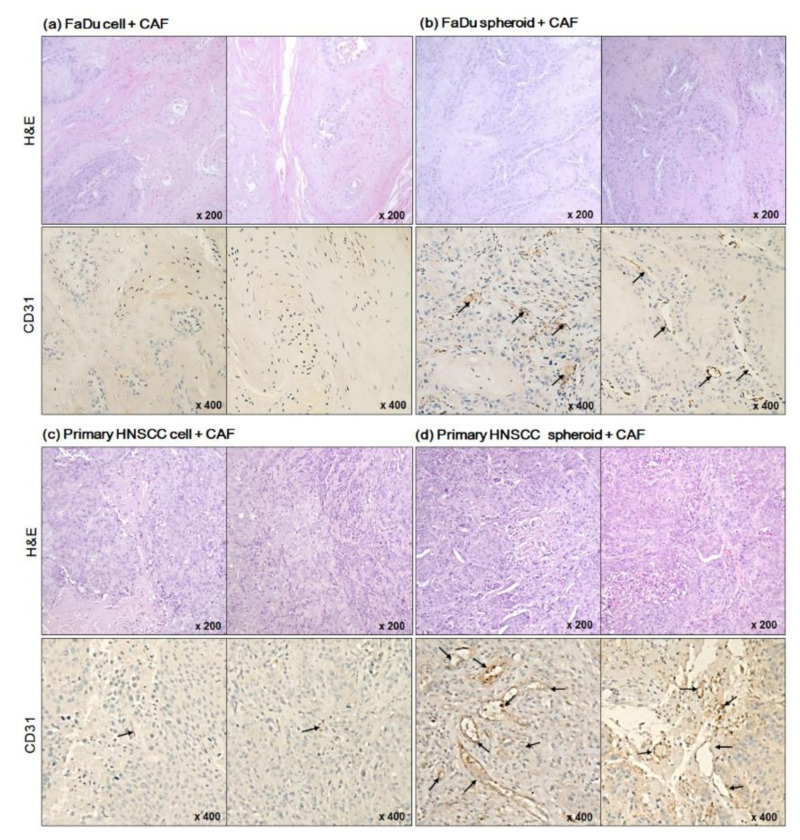

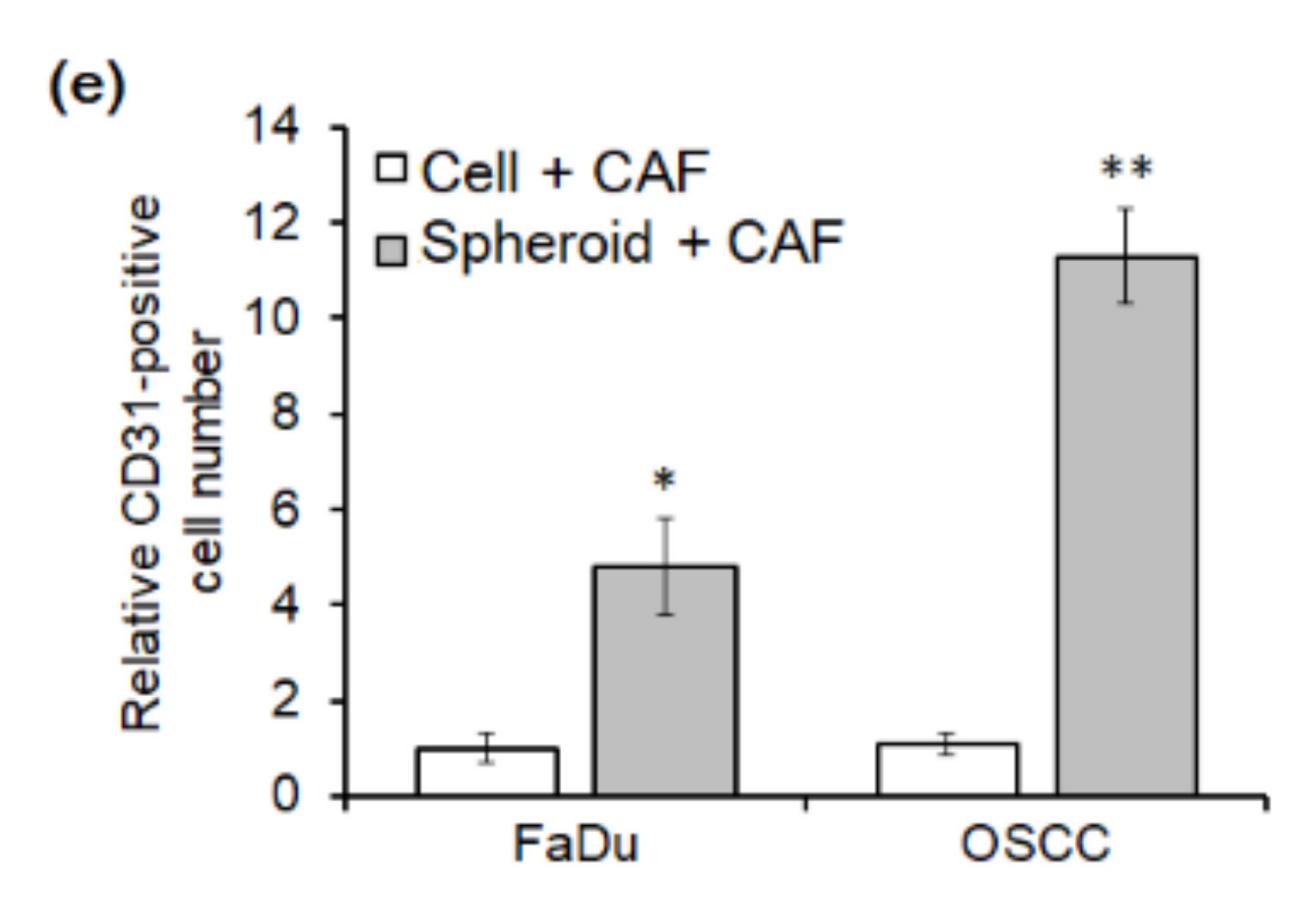
Immunohistochemical analysis of mice tumor tissues stained with anti-CD31 antibody, a specific endothelial marker (**a**–**d**). Representative CD31-positive cells were indicated with black arrows. (**e**) The CD31-positive cells were counted in immunostained mice tumor tissues (* *p* < 0.05, ** *p* < 0.01).

To further evaluate blood vessel formation in mice tumors, we performed IHC with the anti-CD31 antibody. As shown in Figure 6a,c, mice tumors derived from dissociated cells showed no or little CD31-positive cells. However, anti-CD31 staining produced signals in a wide range of tissues derived from FaDu or primary HNSCC spheroids (Figure 6b,d). When we count the CD31-positive cells, a significant difference was found between tumors derived from monolayer cells and spheroids of both FaDu and primary HNCC cells (Figure 6e).

### 2.5. Effect of Exosomes on the mRNA Expression of Angiogenesis-Related Genes in HNSCC Cells

**Figure 7:**
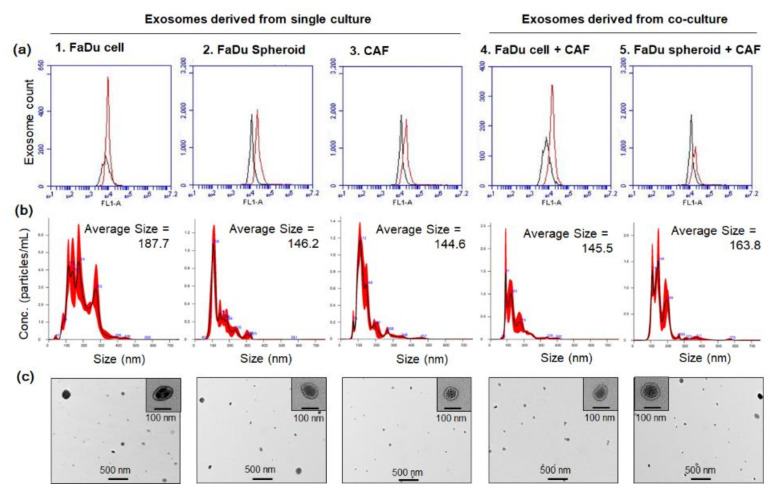
Characterization of exosomes derived from conditioned medium of FaDu cell or spheroid with or without CAF co-culture. (**a**) Exosomes were bound to the magnetic beads for CD63 capture, followed by FACS analysis. (**b**) Exosomes were analyzed for particle size and concentration by the Nanoparticle tracking analysis system using the Nanosight device. (**c**) TEM image of exosomes was defined as round-shaped vesicles. Exosomes were isolated twice under biologically independent conditions, and the representative images are shown.

We evaluated the effect of exosomes isolated from FaDu cell or spheroid under single culture or co-culture with CAF on angiogenesis-related gene expression in FaDu and primary HNSCC cells. As shown in Figure 7a, the purified extracellular vesicles were identified as CD63-positive exosomes. The average size and concentration of exosomes are shown in Figure 7b and Table 1. Figure 7c shows the representative TEM image of each exosome sample. The concentration of exosomes isolated from CAF was significantly lower than that of exosomes isolated from cancer cells or spheroids. In addition, the concentration of exosomes isolated from FaDu and CAF co-culture media was significantly higher than that of FaDu single culture. Interestingly, the concentration of exosomes isolated from FaDu spheroid + CAF co-culture was significantly higher compared to FaDu cell + CAF.

**Figure 8:**
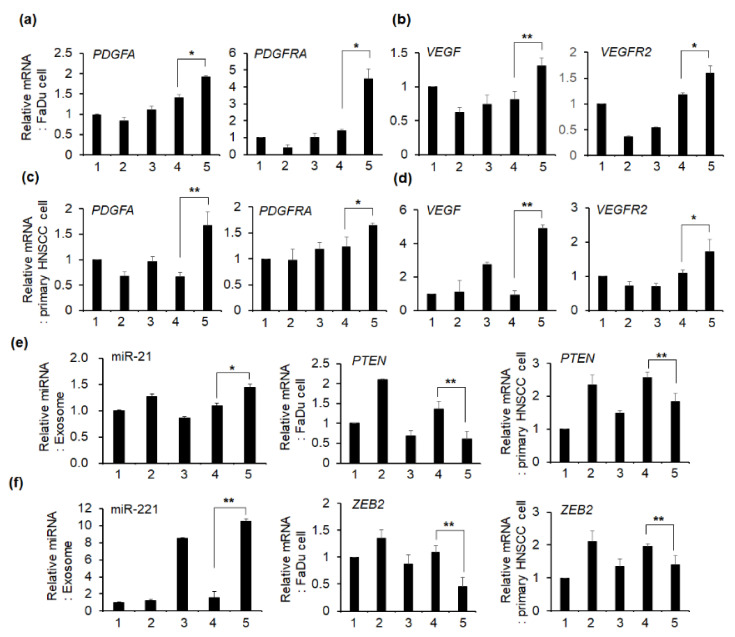
Effect of tumor-derived exosomes on the mRNA expression (fold change) of angiogenesis-related genes in HNSCC cells. (**a**–**d**) After treating each exosome isolated from CMs (1–5) with the same concentration for 48 h, FaDu and primary HNSCC cell mRNA was extracted and qPCR analysis was performed. (**e**,**f**) After extracting miRNA from exosomes isolated from CMs, miR-21 and miR-221 expression levels were analyzed using TaqMan MicroRNA Assay Kit. mRNA expression of *PTEN* and *ZEB2*, direct target genes of miR-21 and miR-221, respectively, was compared in HNSCC cells treated with exosomes for 48 h. PCR analysis was performed two times in triplicate; representative data are shown. The fold change of each mRNA with the mean ± standard deviation is presented (* *p* < 0.05, ** *p* < 0.01). The number of CM from which the exosomes are isolated is as follows: 1, FaDu cell; 2, FaDu spheroid; 3, CAF; 4, FaDu cell + CAF; 5, FaDu spheroid + CAF.

The results of PCR analysis after treating the same number of exosomes in FaDu and primary HNSCC cells are shown in Figure 8. Platelet-derived growth factor A (*PDGFA*) and platelet-derived growth factor receptor A (*PDGFRA*) showed no remarkable difference in the fold change of mRNA expression in cells treated with exosomes isolated from a single culture of FaDu cell, FaDu spheroid, or CAF (Figure 8a,c). However, the treatment with co-culture-derived exosomes induced remarkably higher mRNA expression of *PDGFA* and *PDGFRA* than that with single culture-derived exosomes (Figure 8a,c). In addition, mRNA expression of these two genes was significantly increased when the exosomes derived from CM of FaDu spheroid + CAF co-culture were treated as compared to that of FaDu cell + CAF co-culture (Figure 8a,c). The mRNA expression of vascular endothelial growth factor (*VEGF*) and vascular endothelial growth factor receptor 2 (*VEGFR2*) showed a similar pattern to that of *PDGFA* and *PDGFRA* (Figure 8b,d).

We also analyzed representative exosomal miRNAs regulating angiogenesis in cancer. As shown in Figure 8e,f, miR-21 and miR-221 levels are higher in exosomes derived from CM of FaDu spheroid + CAF co-culture compared to that of FaDu spheroid single culture or FaDu cell + CAF co-culture. *PTEN* [26] and zinc finger E-box-binding homeobox 2 (*ZEB2*) [27], which are direct target genes of miR-21 and miR-221, respectively, are significantly downregulated in HNSCC cells treated with exosomes derived from FaDu spheroid + CAF co-culture compared to exosomes derived from FaDu cell + CAF co-culture or other single culture. Interestingly, mRNA expression of *VEGF* and miR-221 was higher in the exosomes derived from CAF single culture as compared to that of FaDu cell or spheroid single culture. In addition, mRNA expression of these two genes was higher in the exosomes derived from the CAF single culture than in the exosome derived from the FaDU cell + CAF co-culture, suggesting that CAF might play an important regulatory role for angiogenesis in the cancer microenvironment.

## 3. Discussion

To evaluate in vivo tumor models for chemotherapeutic screening under standardized conditions, mouse tumors formed from cells grown as 2D monolayers or 3D spheroids with primary CAFs were characterized. We compared tumorigenicity and blood vessel formation in mice xenograft. Our study revealed that co-injection of patients’ CAFs with 3D spheroids caused not only vigorous tumor formation but also substantial blood vessel formation inside the tumor, with little necrosis, in contrast to the 2D monolayer cells. A previous study showed that intraperitoneal administration of fibroblasts in nude mice facilitates their extensive recruitment into the stromal region of remote subcutaneous tumors derived from human carcinoma cells [28]. In our results, human-specific Ku80 staining in mice tumor tissues formed by cancer cells and patient-derived CAF co-injection shows relatively even distribution in fibroblasts as well as in cancer cells. Therefore, it is reasonable to assume that CAFs are distributed in the tumor mi-croenvironment when co-injected with FaDu, thus contributing to mice xenograft formation. Additionally, fibroblasts are a key player in tumor microenvironment-mediated drug resistance [29,30]. Therefore, the use of 3D spheroids co-injected with patient-derived CAFs to establish a mouse model for drug screening may help to determine a more optimal, personalized chemotherapy regimen.

Hypoxia and necrosis reportedly play important roles in chemotherapy resistance and tumor progression [31,32]. Previous data showed that the development of a necrotic core in cancer patients is correlated with increased tumor size and poor prognosis, such as the emergence of chemoresistance [6,7]. One of the unique clinicopathological characteristics of OSCC tumors is that necrosis is observed only in limited cases [8,9]. Therefore, *in vivo* animal models resulting in very little or no tumor necrosis may be useful for OSCC chemotherapy screening. Considering these points, a mouse model developed from 3D spheroids and CAFs may prove useful as a tool for more standardized anticancer drug screening in the treatment of oral cancer. Our data showing that only the spheroid-derived tumors were significantly reduced in volume upon cisplatin treatment in the mouse tumor model derived from FaDu or primary OSCC cells also support this. Using an in vitro 3D lung cancer model, Lee et al. showed that fibroblasts promoted the formation of nascent vessel-like tubular structures, resulting in the formation of vascularized tumor tissue [33]. Additionally, fibroblasts altered cancer cell gene expression to enhance metastasis and angiogenesis. Although the presence of VEGF was sufficient to induce angiogenesis, it was not sufficient to maintain blood vessels supporting tumor growth [34]. Furthermore, VEGF was expressed in tumor cells, whereas angiopoietin-1 and -2, which promote vascular stability and functionality, were expressed in infiltrating host stromal cells [34]. Therefore, exit from the tumor’s dormant state was marked by stromal cell infiltration, particularly myofibroblasts. However, little is known about tumorigenesis and angiogenesis according to CAFs or cell types that induce xenograft formation in vivo.

The differential blood vessel formation inside mouse tumors in this study, particularly with the 3D spheroids, can be partly explained with tumor-derived exosomes. Exosomes are an important component of the tumor microenvironment [35]. Recently, exosomes have been reported to play an important role in the crosstalk between CAFs and cancer cells, thereby contributing to carcinogenesis and tumor microenvironment [36]. Yang et al. suggested that specific exosomes released from CAFs can be internalized by cancer cells and contribute to progression and metastasis by transferring various types of substances [36]. Correspondingly, the exosomes released by cancer cells can also promote the transformation of CAFs [36]. Cancer cell-derived exosomes have been shown to play a key role in tumor progression by accelerating angiogenesis [37]. For example, exosomes secreted by tumor cells carry several potent pro-angiogenic factors such as *PDGF*, *VEGF*, *TGFβ*, and *bFGF*, which mediate angiogenic activities of endothelial cells [38,39]. In addition, it is well known that exosomal miRNAs serve a crucial role in tumor–endothelial crosstalk, thereby regulating angiogenesis and cancer progression. Hsieh et al. demonstrated that HNSCC-derived exosomes enriched in miR-21 promote tumor progression by facilitating angiogenesis and tumor invasiveness [40]. In the present study, exosomes isolated from CM of HNSCC cell spheroid and CAF co-culture caused remarkably higher expression of genes that promote angiogenesis in HSNCC cells than exosomes obtained from CM of FaDu cell and CAF co-culture. Consistent with these results, a recent paper showed that exosomes derived from 3D marrow stem cells had stronger effects on HUVEC cell proliferation, migration, tube formation, and in vivo angiogenesis compared with 2D-derived exosomes [41]. How the cancer cells secret exosomes with differential functions when in the spheroid or dissociated cell state demands further study. Furthermore, the specific substances that promote angiogenesis among exosome components need to be identified.

There are no more previous studies related to in vivo angiogenesis according to the cancer microenvironment, and in vivo growth of 3D spheroid has not yet been fully characterized. The physicochemical context of 3D spheroids under in vitro or in vivo conditions has recently begun to be elucidated. Moreover, cellular biochemistry is profoundly influenced by the microenvironment, including the extracellular matrix, cell–cell contacts, cell–matrix interactions, cell polarity, and oxygen profiles. Therefore, more in-depth research is needed to evaluate how 3D spheroids of HNSC cells induce vigorous angiogenesis in the mice xenograft model. Above all, it must be determined whether these results can be reproduced with other tumor types. Nevertheless, these results will help identify the molecular mechanisms involved in tumor angiogenesis.

## 4. Materials and Methods

### 4.1. Chemicals and Reagents

Dulbecco’s Modified Eagle’s Medium (DMEM), fetal bovine serum (FBS), and penicillin–streptomycin were acquired from Invitrogen (Carlsbad, CA, USA). Qiazol was purchased from Qiagen (Cat# 79306, Germantown, MD, USA), and PCR Master Mix was purchased from Takara Bio (Otsu, Japan). Rabbit anti-α-SMA (abcam, ab5694) antibody was from Abcam (Cambridge, UK). Anti-FN1 (Proteintech Cat# 66042-1-Ig, RRID:AB_11182385) antibody was from Proteintech (Rosemont, IL, USA). Rabbit anti-CD31 and mouse anti-pan-cytokeratin antibodies were purchased from Abcam (Cambridge, MA, USA). Anti-mouse (Alexa Fluor 488 conjugate) and anti-rabbit (Alexa Fluor 633 conjugate) secondary antibodies were obtained from Invitrogen (Carlsbad, CA, USA). Rabbit anti-Ku80 (Cell Signaling TECHNOLOGY Cat# 2753) antibody was from CST (Cell Signaling TECHNOLOGY, Danvers, MA, USA).

### 4.2. Fibroblast Primary Culture from Fresh HNSCC Tissues

Tumor tissues for fibroblast culture were obtained by surgical resection from two HNSCC patients (patient 1 for CAF1, T4N0M0, gingiva; patient 2 for CAF2, T2N0M0, right lateral border of the tongue) at Kyungpook National University Hospital. Neither of the patients had undergone chemoradiotherapy before surgery. The stroma adjacent to the cancer was carefully separated by a pathologist, cut into the smallest possible pieces in sterile DMEM, and seeded into 10-cm culture dishes supplemented with 10% FBS. After 2–3 weeks, fibroblast cells were cultured in a 6-well plate with a cover slide for immunocytochemical analysis. On the following day, the cells were washed and immediately fixed in 4% paraformaldehyde for 1 h. After washing, cells were blocked with albumin serum for 1 h at room temperature. Cells were immunostained with primary antibody for 16 h at 4°C, followed by incubation with fluorescent secondary antibody at room temperature. Fluorescence images were observed under a fluorescence microscope (Carl Zeiss, Thornwood, NY, USA). To further evaluate the primary CAF cells, the immunostained cells were analyzed using a flow cytometer (BD Accuri C6 Plus, San Jose, CA, USA).

### 4.3. FaDu and Primary oral Squamous Carcinoma Cell Culture and Spheroid Formation

The FaDu human HNSCC cell line (ATCC Cat# HTB-43, RRID: CVCL_1218) was obtained from ATCC (American Type Culture Collection, Manassas, VA, USA) and cultured in DMEM containing 10% FBS and 1% penicillin–streptomycin solution at 37 °C in a 5% CO_2_ humidified atmosphere. The cell line was tested for contamination every 2 months with the CellSafe Mycoplasma PCR detection kit (Cat# CS-D, CellSafe Co., Yongin city, Korea). In addition, to compare the anticancer effect of cisplatin on tumors in the nude mice, we established primary cells from HNSCC patient tumor tissue (T4N0M0, left buccal mucosa). Primary cells were obtained after 4 weeks in culture as adherent colonies in DMEM medium with 5% FBS. To obtain spheroids, cells after 3 passages were seeded into a 96-well U-bottom Ultra-Low Attachment plate (4000 cells/well) (Corning Inc., Tewksbury, MA, USA) and cultured for 2–3 days until spheroid formation (approximately 400 μm in diameter). When we analyzed the spheroid size with Cell^3^ iMager (Screen Holdings, Shiga, Japan), the diameter and surface volume of each spheroid were the same within the error range of 5%. After treatment with trypsin for the spheroid, the number of cells constituting the spheroid was counted.

### 4.4. Mouse Xenograft Model

All experimental protocols with mice followed the ARRIVE guidelines (Animal Research: Reporting of In Vivo Experiments) and were approved by the Animal Ethics Committee of Kyungpook National University (2017-94-2). With respect to tumor size, we followed the guidelines for sacrificing when the mouse weight is reduced by 20% or the tumor volume is 10 cm^3^ or more. Nude mice (6-week-old female BALB/c mice, Hyochang Science, Daegu, Korea) were used to evaluate mouse xenograft derived from FaDu monolayer cells or spheroids. The in vivo tumorigenicity induced by FaDu monolayer cells and 3D spheroids with primary CAFs was compared in the same mice to reduce inter-animal variability. A total of 50 spheroids from cancer cells (approximately 5 × 10^5^ cells) were mixed with primary CAF cells (5 × 10^5^) and immediately injected into the left cheek of mice using a 22-gauge needle. Dissociated cancer cells (5 × 10^5^) were also co-injected with CAF cells (5 × 10^5^) into the oral mucosa of the right cheeks using a 26½-gauge needle. Two different primary CAF cells (CAF1 or CAF2) from passage numbers between 5 and 7 were used, respectively. From 20 days after cell transplantation, tumor size was measured using a caliper. Mice were sacrificed after 7 weeks. Mouse blood vessels were stained by adopting the cardiac perfusion approach prior to sacrifice. Tumor tissue was extracted and separated into two pieces, which were subjected to vessel imaging and tumor histological analysis, respectively. To evaluate whether mice tumor xenograft tissues are derived from injected human cells or not, tumor tissues were stained with Ku80. Ku80 is human-specific and broadly expressed throughout the human body with no or low cross-reactivity toward rat or mouse tissues [42]. Each group comprised five mice.

### 4.5. Tissue Clearing and Blood Vessel Staining

Mice were anesthetized using a rodent inhalation anesthesia apparatus (VetEquip, Livermore, CA, USA), which was equipped with vaporizers for isoflurane. For blood vessel staining, mice received intra-cardiac injections with 100 μL lycopersicon esculentum lectin along with perfusion via the left ventricle aorta. After rinsing with approximately 30 mL PBS for 5 min, mouse tissues were fixed with 50 mL of 4% paraformaldehyde for 30 min. Tumor tissue was removed and placed in 4% paraformaldehyde for 12 h. Tissue (5 mm × 5 mm) clearing was performed using the Binaree Tissue Clearing Kit (SHBC-001, Binaree, Daegu, Korea). Briefly, the tissue was immersed in fixing solution for 24 h followed by immersion in tissue clearing solution and incubated in an incubator shaker at 35°C for 18 h. After rinsing three times with washing solution, the tissue was incubated in Mounting and Storage solution (Binaree, Daegu, Korea) for 24 h.

Blood vessel formation in tumor tissues was evaluated using a Lightsheet Z.1 fluorescence microscope (LSFM, Zeiss Corporation, Jena, Germany) with 5 ×/0.1 dual side illumination optic and a Plan-Neofluar 5 ×/0.16 objective lens (Zeiss). Fluorescence was excited with lasers at wavelengths of 488 nm and 561 nm, and emission was detected using 505–545 nm and 575–615 nm band-pass filters. The LSFM imaging data were saved as a czi file using the ZEN software (Zeiss), and image reconstruction was subsequently performed using Arivis Vision4D software (arivis-AG, Rostock, Germany) and Imaris software (Bitplane, Concord, MA, USA).

### 4.6. Real-Time Polymerase Chain Reaction (qPCR) of mRNA and miRNA

RNA extraction, cDNA synthesis, and gene expression normalization were performed according to standard protocols [43]. The primers employed in qPCR are listed in Supplementary Table S1. qPCR was performed using an ABI 7500 real-time PCR system (Applied Biosystems, Waltham, MA, USA). Expression levels of these genes were normalized to those of GAPDH. The fold change of gene expression level was calculated based on the values of the Δcycle threshold (ΔCt), which was determined by normalizing the average Ct value of each treatment to that of the endogenous GAPDH control and then calculating the 2^−ΔΔCt^ value for each treatment. miRNAs were extracted using a miRNeasyR^®^ Mini Kit (Qiagen, Germantown, MD, USA), according to the manufacturer’s instructions. cDNA was synthesized using RT-PCR Kit (Cat# 436596, Applied Biosystems, Waltham, MA, USA) with 10 ng of miRNA. miRNA level was quantified using a TaqMan MicroRNA Assay Kit (Applied Biosystems, Waltham, MA, USA).

### 4.7. Cisplatin Effect on Mice Tumor Derived from FaDu and HNSCC Primary Cell

To compare the effect of cisplatin, one of the representative chemotherapeutics for cancer, on tumors derived from dissociated cells or spheroids, a xenograft nude mouse model was established from FaDu or HNSCC patient-derived primary cancer cells. A total of 50 spheroids from cancer cells (approximately 5 × 10^5^ cells) were mixed with primary CAF2 cells (5 × 10^5^) and immediately injected into the oral mucosa of both mouse cheeks using a 22-gauge needle. Dissociated cancer cells (5 × 10^5^) were also co-injected with CAF cells (5 × 10^5^) into the oral mucosa of both cheeks. The spheroid group and the dissociated cell group consisted of eight mice each with transplantation on both cheeks. After tumor formation, the groups were divided into two groups of cisplatin injection and PBS vehicle injection control. After tumor formation was detected, mice were divided into two groups. Cisplatin (2.5 mg/kg) or vehicle was intraperitoneally injected 3 times a week for 18–23 days, and mice were sacrificed.

### 4.8. Exosome Isolation and Characterization

FaDu spheroids or cells were co-cultured with primary CAF2 in DMEM containing 10% FBS and 1% penicillin–streptomycin solution using Transwell (Corning, Tewksbury, MA, USA). The proportion of FaDu cells and CAFs in both 2D and 3D co-culture is 50% and 50%. The sum of the total number of cells in both monoculture and co-culture was 2 × 10^6^. After 24 h, the growth medium was replaced with serum-free DMEM and maintained for 8 h more. The conditioned medium (CM) was collected for exosome isolation. Exosomes were isolated by ultracentrifugation in accordance with the manufacturer’s instructions. Briefly, the CM was centrifuged at 2000× *g* for 30 min at 4 °C. The supernatant was then transferred to a new tube and combined with Total Exosome Isolation reagent (Invitrogen, Thermo Fisher Scientific, Waltham, MA, USA) and incubated overnight according to the manufacturer’s protocol. The extracellular vesicle was then isolated by centrifugation at 10,000× *g* at 4 °C for 1 h. The exosome-containing pellets were re-suspended in PBS solution. The yield of the exosome preparation was determined by the Nanoparticle tracking analysis system (Nanosight NS300, Malvern, United Kingdom). CD63 was used as specific exosome markers. To characterize the isolated exosomes, they were bound to the magnetic beads for CD63 capture (CD63 Exo-Flow Capture Kit, EXOFLOW300A-1, System Biosciences, Palo Alto, CA, USA), followed by FACS analysis (Accuri™ C6 Plus Flow Cytometer, Becton Dickinson and Company, Franklin Lakes, NJ, USA). To acquire the TEM image of the exosome, we prepared three grids (200 mesh, 01800-F, Ted Pella Inc., Redding, CA., USA.) coated with Formvar/carbon per sample. Grids were then air-dried and viewed on a Bio transmission electron microscope (Hitachi TEM HT7700, Tokyo, Japan).

### 4.9. Effect of Exosome on the mRNA Expression Related to Blood Vessel Formation in Cancer Cells

FaDu and primary HNSCC cells were seeded at 300,000 cells/well in 6-well plates. Cell culture was performed under serum-free DMEM for 16 h before the treatment of exosome. Exosome was treated at 11 × 10^5^ particle/well to HNSCC cells for 48 h, followed by mRNA extraction for qPCR analysis.

### 4.10. Statistical Analysis

Statistical parameters, including in vivo animal analysis, are indicated in the figure legends. All statistical analysis was conducted with Origin v.8.0 software (OriginLab, Northampton, MA, USA). Differences between groups were evaluated with the parametric two-tailed unpaired Student’s *t*-test. Data were considered significant when *p* < 0.05. Significant *p* values are represented in the figures.

## 5. Conclusions

Our results reveal that tumor tissue characteristics differ with the injection of cells grown as a 2D monolayer or 3D spheroids with CAF co-injection. The mouse xenograft tumor model derived from 3D spheroids of cancer cells with co-injection of patient-derived CAFs showed a remarkable extent of angiogenesis inside the tumor. In addition, cisplatin injection into the mice with cancer cell- or spheroid-derived tumors caused a significant decrease in tumor size only in the spheroid-derived tumors, supporting our hypothesis. This data should be helpful in reproduce more accurately the cancer microenvironment, particularly in HNSCC, thereby facilitating a more optimal evaluation of drug effects in mice xenograft. Additionally, CAFs derived from HNSCC patients are expected to more accurately replicate the patient’s specific cancer microenvironment in mouse models, so it is expected to be applied to experimental models for patient-specific drug treatment.

## Figures and Tables

**Table 1:** Exosome concentration.

Exosome Isolated from		Concentration (Particles/mL/2 × 10^6^ Cells)	*p*-Value	
Single culture	CAF	1.50 × 10^8^ ± 1.88 × 10^7^		
	FaDu cell	6.29× 10^8^ ± 4.78 × 10^7^	*p* > 0.05	*p* < 0.05
	FaDu spheroid	5.47 × 10^8^± 8.84 × 10^7^		
Co-culture	FaDu cell + CAF	8.21 × 10^8^± 9.52 × 10^7^	*p* < 0.05	
	FaDu spheroid + CAF	11.1 × 10^8^ ± 1.13 × 10^7^		

## Data Availability

The raw/processed data required to reproduce these findings are available from the corresponding author on reasonable request.

## Supplementary Materials

The following are available online at https://www.mdpi.com/article/10.3390/ijms22158245/s1.

## References

[1] Parkin D.M., Bray F., Ferlay J., Pisani P. (2005). Global Cancer Statistics, 2002. CA Cancer J. Clin..

[2] Galitis E., Droukas V., Tzakis M., Psarras V., Galiti D., Kyrodimos E., Trichas M., Psyrri A., Papadogeorgakis N., Kouri M. (2017). Trismus and Reduced Quality of Life in Patients With Oral Squamous Cell Carcinoma, Who Received Post-Operative Radio-Therapy Alone or Combined With Chemotherapy. J. Clin. Oncol..

[3] Maggiore R., Zumsteg Z.S., BrintzenhofeSzoc K., Trevino K.M., Gajra A., Korc-Grodzicki B., Epstein J.B., Bond S.M., Parker I., Kish J.A. (2017). The Older Adult With Locoregionally Advanced Head and Neck Squamous Cell Carcinoma: Knowledge Gaps and Future Direction in Assessment and Treatment. Int. J. Radiat. Oncol. Biol. Phys..

[4] De Sousa E.M.F., Vermeulen L., Fessler E., Medema J.P. (2013). Cancer Heterogeneity–A Multifaceted View. EMBO Rep..

[5] Albini A., Sporn M.B. (2007). The Tumour Microenvironment as a Target for Chemoprevention. Nat. Rev. Cancer.

[6] Tomes L., Emberley E., Niu Y., Troup S., Pastorek J., Strange K., Harris A., Watson P.H. (2003). Necrosis and Hypoxia in Invasive Breast Carcinoma. Breast Cancer Res. Treat..

[7] Lekshmi A., Varadarajan S.N., Lupitha S.S., Indira D., Mathew K.A., Nair A.C., Nair M., Prasad T., Sekar H., Gopalakrishnan (2017). A Quantitative Real-Time Approach for Discriminating Apoptosis and Necrosis. Cell Death Discov..

[8] Ismerim A.B., Xavier F.C.A., Cangussu M.C.T., Ramalho L.M.P., Agra I.M.G., Santos J.N.D. (2016). Useful Histological Findings in Incisional Biopsies of Oral Squamous Cell Carcinoma. Srp. Arh. Celok. Lek..

[9] Pires F.R., Ramos A.B., Oliveira J.B., Tavares A.S., Luz P.S., Santos T.C. (2013). Oral Squamous Cell Carcinoma: Clinicopathological Features From 346 Cases From a Single Oral Pathology Service During an 8-Year Period. J. Appl. Oral. Sci..

[10] Tung Y.C., Hsiao A.Y., Allen S.G., Torisawa Y.S., Ho M., Takayama S. (2011). High-Throughput 3D Spheroid Culture and Drug Testing Using a 384 Hanging Drop Array. Analyst.

[11] Hirschhaeuser F., Leidig T., Rodday B., Lindemann C., Mueller-Klieser W. (2009). Test System for Trifunctional Antibodies in 3D MCTS Culture. J. Biomol. Screen..

[12] Massa A., Varamo C., Vita F., Tavolari S., Peraldo-Neia C., Brandi G., Rizzo A., Cavalloni G., Aglietta M. (2020). Evolution of the Experimental Models of Cholangiocarcinoma. Cancers.

[13] Jung H.R., Kang H.M., Ryu J.W., Kim D.S., Noh K.H., Kim E.S., Lee H.J., Chung K.S., Cho H.S., Kim N.S. (2017). Cell Spheroids with Enhanced Aggressiveness to Mimic Human Liver Cancer In Vitro and In Vivo. Sci. Rep..

[14] Bhowmick N.A., Neilson E.G., Moses L.H. (2004). Stromal Fibroblasts in Cancer Initiation and Progression. Nature.

[15] Carmeliet P., Jain R.K. (2000). Angiogenesis in Cancer and Other Diseases. Nature.

[16] Sadlonova A., Bowe D.B., Novak Z., Mukherjee S., Duncan V.E., Page G.P., Frost A.R. (2009). Identification of Molecular Distinctions Between Normal Breast-Associated Fibroblasts and Breast Cancer-Associated Fibro-Blasts. Cancer Microenviron..

[17] Suetsugu A., Osawa Y., Nagaki M., Saji S., Moriwaki H., Bouvet M., Hoffman R.M. (2011). Identification of Molecular Distinctions Between Normal Breast-Associated Fibroblasts and Breast Cancer-Associated Fibro-Blasts. J. Cell. Biochem..

[18] Olumi A.F., Grossfeld G.D., Hayward S.W., Carroll P.R., Tlsty T.D., Cunha G.R. (1999). Carcinoma-Associated Fibroblasts Direct Tumor Progression of Initiated Human Prostatic Epithelium. Cancer Res..

[19] Peltanova B., Liskova M., Gumulec J., Raudenska M., Polanska H.H., Vaculovic T., Kalfert D., Grega M., Plzak J., Betka J. (2021). Sensitivity to Cisplatin in Head and Neck Cancer Cells Is Significantly Affected by Patient-Derived Cancer-Associated Fi-Broblasts. Int. J. Mol. Sci..

[20] Liao J.K., Zhou B., Zhuang X.M., Zhuang P.L., Zhang D.M., Chen W.L. (2018). Cancer-Associated Fi Broblasts Confer Cisplatin Resistance of Tongue Cancer via Autophagy Activation. Biomed. Pharmacother..

[21] Dai J., Su Y., Zhong S., Cong L., Liu B., Yang J., Tao Y., He Z., Chen C., Jiang Y. (2020). Exosomes: Key Players in Cancer and Potential Therapeutic Strategy. Signal Transduct. Target. Ther..

[22] Mimeault M., Batra S.K. (2014). Molecular Biomarkers of Cancer Stem/Progenitor Cells Associated With Progression, Metastases, and Treatment Resistance of Aggressive Cancers. Cancer Epidemiol. Biomark. Prev..

[23] Ludwig N., Yerneni S.S., Razzo B.M., Whiteside T.L. (2018). Exosomes from HNSCC Promote Angiogenesis through Reprogramming of Endothelial Cells. Mol. Cancer Res..

[24] Qin X., Guo H., Wang X., Zhu X., Yan M., Wang X., Xu Q., Shi J., Lu E., Chen W. (2019). Exosomal miR-196a Derived From Cancer-Associated Fibroblasts Confers Cisplatin Resistance in Head and Neck Cancer Through Targeting CDKN1B and *ING5*. Genome Biol..

[25] Micke P., Ostman A. (2005). Exploring the Tumour Environment: Cancer-Associated Fibroblasts As Targets in Cancer Therapy. Expert. Opin. Ther. Targets.

[26] Liu L.Z., Li C., Chen Q., Jing Y., Carpenter R., Jiang Y., Kung H.F., Lai L., Jiang B.H. (2011). MiR-21 Induced Angiogenesis Through AKT and ERK Activation and HIF-1alpha Expression. PLoS ONE.

[27] Chen Y., Banda M., Speyer C.L., Smith J.S., Rabson A.B., Gorski D.H. (2010). Regulation of the Expression and Activity of the Antiangiogenic Homeobox Gene GAX/MEOX2 by ZEB2 and microRNA-221. Mol. Cell. Biol..

[28] Granot D., Addadi Y., Kalchenko V., Harmelin A., Kunz-Schughart L.A., Neeman M. (2007). In Vivo Imaging of the Systemic Recruitment of Fibroblasts to the Angiogenic Rim of Ovarian Carcinoma Tumors. Cancer Res..

[29] Aref A.R., Huang R.Y., Yu W., Chua K.N., Sun W., Tu T.Y., Bai J., Sim W.J., Zervantonakis I.K., Thiery J.P. (2013). Screening Therapeutic EMT Blocking Agents in a Three-Dimensional Microenvironment. Integr. Biol..

[30] Majety M., Pradel L.P., Gies M., Ries C.H. (2015). Fibroblasts Influence Survival and Therapeutic Response in a 3D Co-Culture Model. PLoS ONE.

[31] Shannon A.M., Bouchier-Hayes D.J., Condron C.M., Toomey D. (2003). Tumour Hypoxia, Chemotherapeutic Resistance and Hypoxia-Related Therapies. Cancer Treat. Rev..

[32] Meijer T.W., Kaanders J.H., Span P.N., Bussink J. (2012). Targeting Hypoxia, HIF-1, and Tumor Glucose Metabolism To Improve Radiotherapy Efficacy. Clin. Cancer Res..

[33] Lee S.W., Kwak H.S., Kang M.H., Park Y.Y., Jeong G.S. (2018). Fibroblast-Associated Tumour Microenvironment Induces Vascular Structure-Networked Tumouroid. Sci. Rep..

[34] Gilad A.A., Israely T., Dafni H., Meir G., Cohen B., Neeman M. (2005). Functional and Molecular Mapping of Uncoupling Between Vascular Permeability and Loss of Vascular Maturation in Ovari-An Carcinoma Xenografts: The Role of Stroma Cells in Tumor Angiogenesis. Int. J. Cancer.

[35] Wang Z., Chen J.Q., Liu J.L., Tian L. (2016). Exosomes in Tumor Microenvironment: Novel Transporters and Biomarkers. J. Transl. Med..

[36] Yang X., Li Y., Zou L., Zhu Z. (2019). Role of Exosomes in Crosstalk Between Cancer-Associated Fibroblasts and Cancer Cells. Front Oncol..

[37] Kalluri R. (2016). The Biology and Function of Exosomes in Cancer. J. Clin. Investig..

[38] Ludwig N., Whiteside T.L. (2018). Potential Roles of Tumor-Derived Exosomes in Angiogenesis. Expert Opin. Ther. Targets.

[39] Giusti I., Monache S.D., di Francesco M., Sanita P., D’Ascenzo S., Gravina G.L., Festuccia C., Dolo V. (2016). From Glioblastoma to Endothelial Cells Through Extracellular Vesicles: Messages for Angiogenesis. Tumour Biol..

[40] Hsieh C.H., Tai S.K., Yang M.H. (2018). Snail-Overexpressing Cancer Cells Promote M2-Like Polarization of Tumor-Associated Macrophages by Delivering MiR-21-Abundant Exosomes. Neoplasia.

[41] Gao W., Liang T., He R., Ren J., Yao H., Wang K., Zhu L., Xu Y. (2020). Exosomes From 3D Culture of Marrow Stem Cells Enhances Endothelial Cell Proliferation, Migration, and Angiogenesis via Activation of the HMGB1/AKT Pathway. Stem Cell Res..

[42] Allard J., Li K., Lopez X.M., Blanchard S., Barbot P., Rorive S., Decaestecker C., Pochet R., Bohl D., Lepore A.C. (2014). Immunohistochemical Toolkit for Tracking and Quantifying Xenotransplanted Human Stem Cells. Regen. Med..

[43] Kim J., Kang H.S., Lee Y.J., Lee H.J., Yun J., Shin J.H., Lee C.W., Kwon B.M., Hong S.H. (2014). EGR1-Dependent PTEN Upregulation by 2-Benzoyloxycinnamaldehyde Attenuates Cell Invasion and EMT in Colon Can-Cer. Cancer Lett..

