# Supplementary material for: Differential Angiogenic Potential of 3-Dimension Spheroid of HNSCC Cells in Mouse Xenograft
Source: Int J Mol Sci. 2021 Jul 31;22(15):8245. doi: 10.3390/ijms22158245 (PMC8348975; doi:10.3390/ijms22158245)
Supplement: Supplementary file 1 [file ijms-22-08245-s001.zip › Suppl data-Table & Figure-2021-0608.pdf]

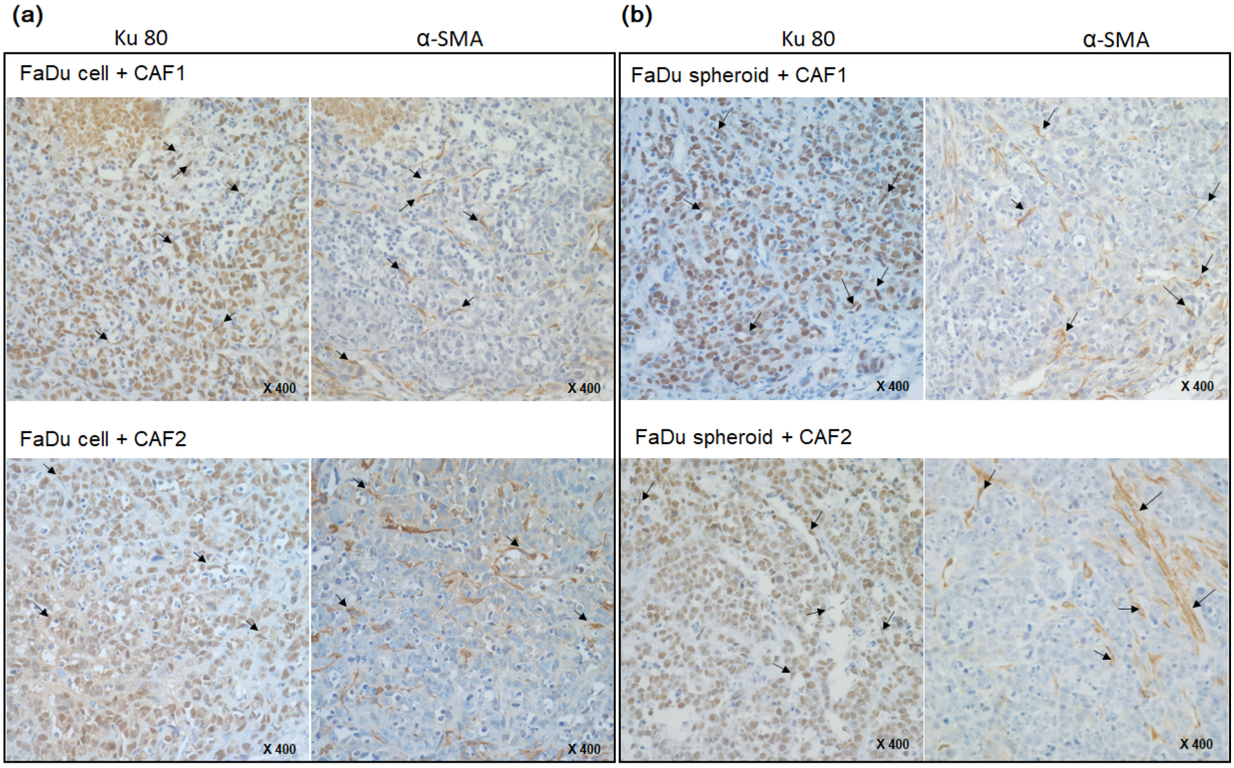

Figure S1. Immunohistochemical analysis of mice xenograft tumors tissues derived from FaDu cells or spheroids with primary CAFs. Tissues were immunostained with anti-Ku80 and α-SMA antibody. Ku80 biomarkers are broadly stained in tumor tissues, such as epithelial cells and fibroblasts. Fibroblast-specific α-SMA staining was also detected inside the epithelial tumor cells.

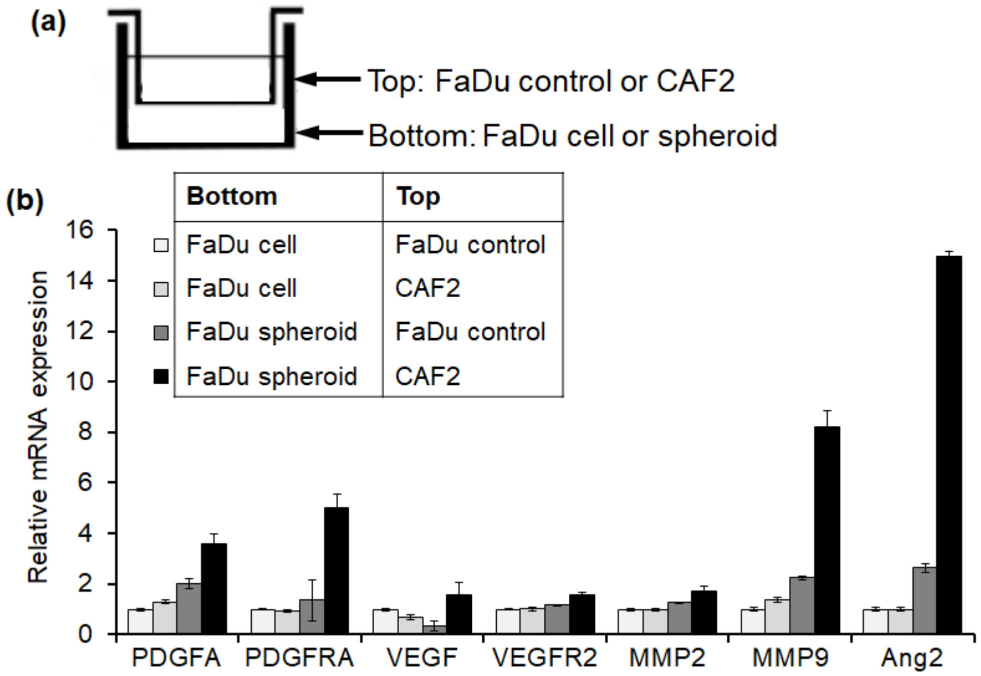

Figure S2. mRNA expression of angiogenesis-related genes in FaDu with or without CAF co-culture. (a) FaDu control or CAF2 cells (3×10^5) were seeded in the Transwell of the 6-well plate. Same number of FaDu cell was added to the bottom well for 2D monolayer or 3D spheroid culture. FaDu spheroid culture was performed in non-adherent 6-well plate. After culturing for 48 h, FaDu cell or spheroid in the bottom well was collected, followed by mRNA extraction for qPCR. Results represent the mean ± standard deviation of 3 experiments.

Video legend

Video 1. Blood vessel image of tumor tissues from the right mouse cheek, which were derived from FaDu monolayer cells co-injected with patient CAF1.

Video 2. Blood vessel image of tumor tissues from the left mouse cheek, which were derived from FaDu spheroids co-injected with patient CAF1.

Video 3. Blood vessel image of tumor tissues from the right mouse cheek, which were derived from FaDu monolayer cells co-injected with patient CAF2.

Video 4. Blood vessel image of tumor tissues from the left mouse cheek, which were derived from FaDu spheroids co-injected with patient CAF2.

Table S1. The forward and reverse primers used for qPCR.

Gene name	F/R	sequence (5'→3')
c-Myc	Forward	ACCCTTGCCGCATCCACGAAAC
	Reverse	CGTAGTCGAGGTCATAGTTCCTGTTGG
Ki-67	Forward	CACCTAAGGAAGAGGCCCAA
	Reverse	TGGTGGAGATTTGCAGGCTA
PCNA	Forward	GCGTGAACCTCACCAGTATG
	Reverse	TCTCCTGGTTTGGTGCTTCA
HMGB1	Forward	TGTCTCCCTAGAGCCCATCT
	Reverse	GACTAGTCAGAACGGGTCGT
TNF-α	Forward	GGCGTGGAGCTGAGAGATAAC
	Reverse	GGTGTGGGTGAGGAGCACAT
PDGFA	Forward	GTGCGGTCTTTGTTCTCCTC
	Reverse	CTTACTGCTTCACCGAGTGC
PDGFRA	Forward	GCGACAAGGTATAATGGCAGAAT
	Reverse	TTGAAGGCAGGCACATTTACA
VEGF	Forward	CTACCTCCACCATGCCAAGT
	Reverse	GCAGTAGCTGCGCTGATAGA
VEGFR2	Forward	CTGACTGCACAAACCAGCTT
	Reverse	ACTTTGACACCACACACAGC
PTEN	Forward	TCAGTGGCGGAACTTGCAA
	Reverse	CATGAACTTGTCTTCCCGTCG
ZEB2	Forward	AGTACCAGCGAAACAAGGA
	Reverse	TTTGCGAGACAGACAGGAGT
MMP2	Forward	GTCCAGAGGCAATGCAGTGGG
	Reverse	TCACTAGGCCAGCTGGTTGGTTC
MMP9	Forward	ACGACGTCTTCCAGTACCGAGA
	Reverse	TAGGTCACGTAGCCCACTTGGT
Ang2	Forward	GCATCAGCCAACCAGGAAAT
	Reverse	TGTGTTCTGCCTCTGTGGAT
GAPDH	Forward	AGATCATCAGCAATGCCTCCTG
	Reverse	CTGGGCAGGGCTTATTCCTTTTCT
